# 

**Published:** 1860-05

**Authors:** 


### CONTENTS.

### ORIGINAL ESSAYS AND COMMUNICATIONS.

Pathology of the Mouth.......... 569
Thoughts on Decay and its Treatment. BY GEO. S FOUKE,.......... 576
Case of Arsenical Poisoning. Reported by G. S. FOUKE,.......... 580
Cleanliness a Preservative of the Teeth.......... 582
Commencement of the Pennsylvania College of Dental Surgeons,.......... 583
Preservation of the Teeth.......... 585
Modes of Practice and their Vicissitudes. BY J. TAFT,.......... 591
Is there Circulation in the Dentine. BY E. COLLINS,.......... 596
Ppreparation and Filling of a Simple Cavity in the Right Superior Cuspid, Posterior Proximal Surface. BY D. J. H. PAINE,.......... 597
Popular Instruction,.......... 6[illegible]0

### PROCEEDINGS OF SOCIETIES.

Proceedings and Discussions of the Cincinnati Dental Association,.......... 602
Mad River Valley Dental Association,.......... 605

SELECTIONS.......... 607

Subjects for Discussion by the Western Dental Society, at its next Meeting,.......... 611
American Dentaf Convention.......... 612

EDITORIAL.......... 613

